# Trigger points of palliative care assessment in inherited metabolic diseases

**DOI:** 10.1186/s13023-025-04128-x

**Published:** 2025-12-29

**Authors:** Gustavo Marquezani Spolador, Rita Tiziana Verardo Polastrini, Ivete Zoboli, Ana Cristina Henrique, Elaine Freitas, Andréa Gislene do Nascimento, Camila Pugliese, Fernando Kok, Clarissa Bueno, Silvia Maria de Macedo Barbosa

**Affiliations:** 1 Department of Pain and Pediatric Palliative Care, University of Sao Paulo, Sao Paulo, 05403-000 Brazil; 2 Department of Neurometabolic Diseases, University of Sao Paulo, Sao Paulo, Brazil; 3 Department of Social Work and Social Care, University of Sao Paulo, Sao Paulo, Brazil; 4 Department of Nutrition and Dietetics, University of Sao Paulo, Sao Paulo, Brazil

**Keywords:** Inborn errors of metabolism, Metabolic diseases, Palliative care, Pediatrics, Metabolism, Pediatric palliative care

## Abstract

The interaction between palliative care (PC) and inherited metabolic diseases (IMDs) is an area of increasing clinical importance. However, the integration of PC in this field remains limited due to several factors, including a lack of understanding of the specialty, reluctance to refer, limited resources and the absence of specific eligibility criteria for IMD patients. This article proposes a novel framework of trigger points to guide timely PC referral in the IMD field. Based on a review of the literature and the pathophysiological characteristics of IMDs, three pairs of illness trajectories were established. The first pair addresses intoxication disorders, comprising Group 1 (organic acidurias, urea cycle disorders and some aminoacidopathies) and Group 2 (metabolite repair defects, amino acids synthesis defects and the fatty acids synthesis defects. The second pair focuses on energy disorders, including Group 1 (fatty acid oxidation dirsorders [FAOD] and membrane transport disorders [MCT/GLUT]) and Group 2 (select mitochondrial disorders). The last pair covers complex molecules disorders, with Group 1 (storage disorders and congenital disroders of glycosylation [CDGs] and Group 2 (cellular processing and trafficking defects). The proposed trigger points for PC referral include the time of diagnosis, periods of prognostic uncertainty, challenges and burdens associated with dietary and treatment management, metabolic crises, the emergence of multimorbidity, adaptation to a “new normal”, and the consideration of new therapies or end-of-life care. This framework provides an opportunity for clinicians to establish a partnership with patients and their families to enhance care by focusing on the patient as the central figure of their illness trajectory.

## Introduction

Palliative care (PC) has emerged as a crucial interdisciplinary specialty, providing symptom management, psychological support, and guidance in the medical decision-making process for various conditions. Reinforcing its core premise of offering early, holistic care, PC has become increasingly relevant in the context of inherited metabolic diseases (IMDs), addressing both the clinical burden and the public health implications of cost effectiveness and improved quality of life [1–3].

The integration of PC in IMD care has increased in the last five years. Few, but robust publications have documented the growing number of IMD patients being followed up by PC teams, highlighting frequent neurological, respiratory and gastrointestinal symptoms. These studies have also provided crucial epidemiological data, including the age of diagnosis, the age of PC referral, and the time elapsed between the two [4, 5]. 

Despite this progress, the seamless and non-stigmatized integration of PC into routine IMD clinical practice remains a challenge. Key barriers include a lack of specialized resources, professional ignorance about PC’s scope, reluctance to refer; reluctance from both clinicians and families to engage in referrals, and restrictive eligibility criteria for specialist PC services [6]. 

To address these obstacles, institutional continuing education and collaborative scientific publications are essential. These initiatives can help to correct misconceptions about PC, fostering a new healthcare framework where the specialty is considered a standard of care.

This article aims to transform the stigma surrounding palliative care assessment by presenting a practical framework of trigger points for metabolic physicians. This framework is designed to serve as a useful tool throughout the patient’s illness trajectory, ensuring that the patient’s holistic needs remain the central focus on care.

## Methods

### Review of articles addressing IMDs in PC

A systematic review of scientific literature was conducted in PubMed, using the search terms “Palliative Care”, “Palliative Medicine”, “Hospice Care”, and “Metabolic Diseases”, “Inborn Errors of Metabolism”, or “Inherited Metabolic Diseases”.v The search strategy was not limited to Medical Subject Headings (MeSH), due to the limited and heterogeneous nature of the existing literature at the intersection of these two fields. Furthermore, many retrieved articles cited, nut did not extensively discuss, PC in the context of IMD.

In this way, our search identified four articles published in the last ten years that address the intersection between PC and IMD. The two earliest publications, from 2018, present a general overview of IMD in PC setting and were the first to highlight the prevalence of these disorders within in specialty. A subsequent 2021 study meticulously examined the profile of IMD in a cohort of hospitalized patients over a two-month period. This study not only identified the most prevalent symptoms in this population but also advocated for the integration of PC to optimize symptom management and promote comprehensive care [4, 7, 8]. 

Finally, the most recent publication presented a global evaluation of IMDs, categorized by their pathophysiological groups. This study established a clear relationship between the time of diagnosis and the subsequent referral to pediatric palliative care (PPC) group [5]. 

### Establishing a parallel between IMD and PC trajectories

A recent and innovative publication proposed an adaptation of PPC frameworks to the specific needs of patients with IMD. This framework considers the pathophysiological groups of IMDs, their most frequent outcomes, and the four illness trajectories established by Hain (2008) for conditions eligible for pediatric palliative care (PPC), as defined by the Association of Children’s Palliative Care [9–11]. 

This proposal, while not encompassing all the IMDs, initiated a discussion regarding the potential for early palliative care integration for patients whose illness trajectories can be recognized [10]. 

### Identifying common trigger points

Regarding the concepts of chronic complex conditions, conditions eligible to PPC, their trajectories, and the adaptation of these to IMDs, we identified common trigger points for the initiation of PC [12].

These trigger points were established based on the pathophysiological groups of IMDs and their main characteristics [9]. Furthermore, we defined a temporal scale for PC referral, which reflects both the World Health Organization (WHO) recommendations and the current clinical practice [1, 13]. 

## Results

Regarding the proposition of IMD trajectories within context of palliative care, we used these trajectories as the foundation for the allocating trigger point.10 Based on the pathophysiological groups of IMDs – namely, intoxication, energy and complex molecules - two categories of trajectories were established to account for the heterogeneous manifestations of each disorder.

**Fig. 1:**
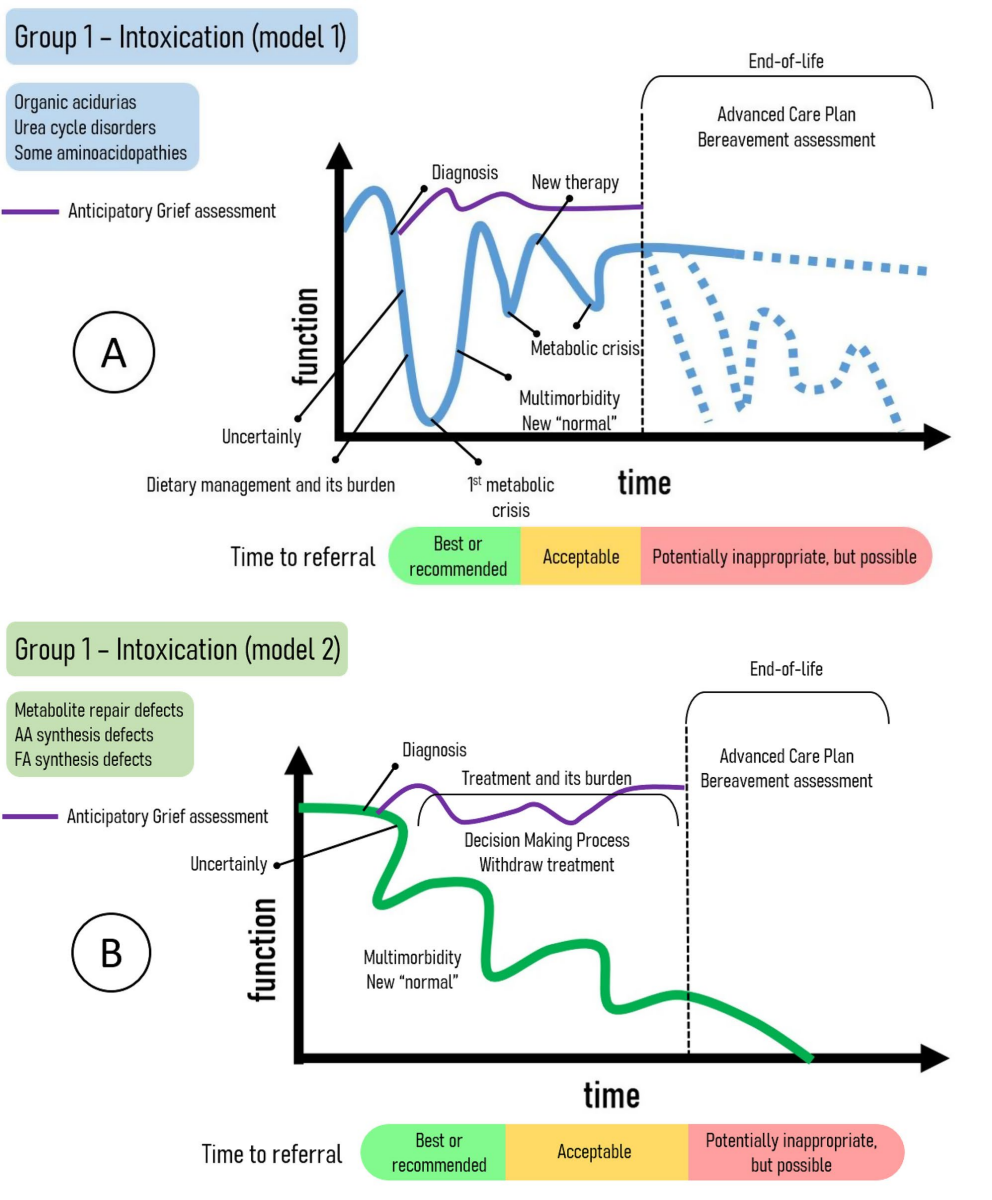
Trajectories of intermediate IMD (IIMD). **A** – The most common IIMD represented by organic acidurias, urea cycle disorders and some of aminoacidopathies. **B** – Some synthesis defects of amino acids and fatty acids and metabolite repair defects present this trajectory

For group 1 (intoxication disorders), category 1 contemplates the most common clinical courses observed in organic acidurias, urea cycle disorders, and certain aminoacidopathies. In contrast, category 2 addresses a different evolutionary pattern, specifically for disorders involving metabolite repair defects, amino acids synthesis defects, and the fatty acids synthesis defects. Figure 1 summarizes the trajectories for intoxication IMDs (IIMDs), highlighting the proposed trigger points and a temporal scale of optimal PC referral.

**Fig. 2:**
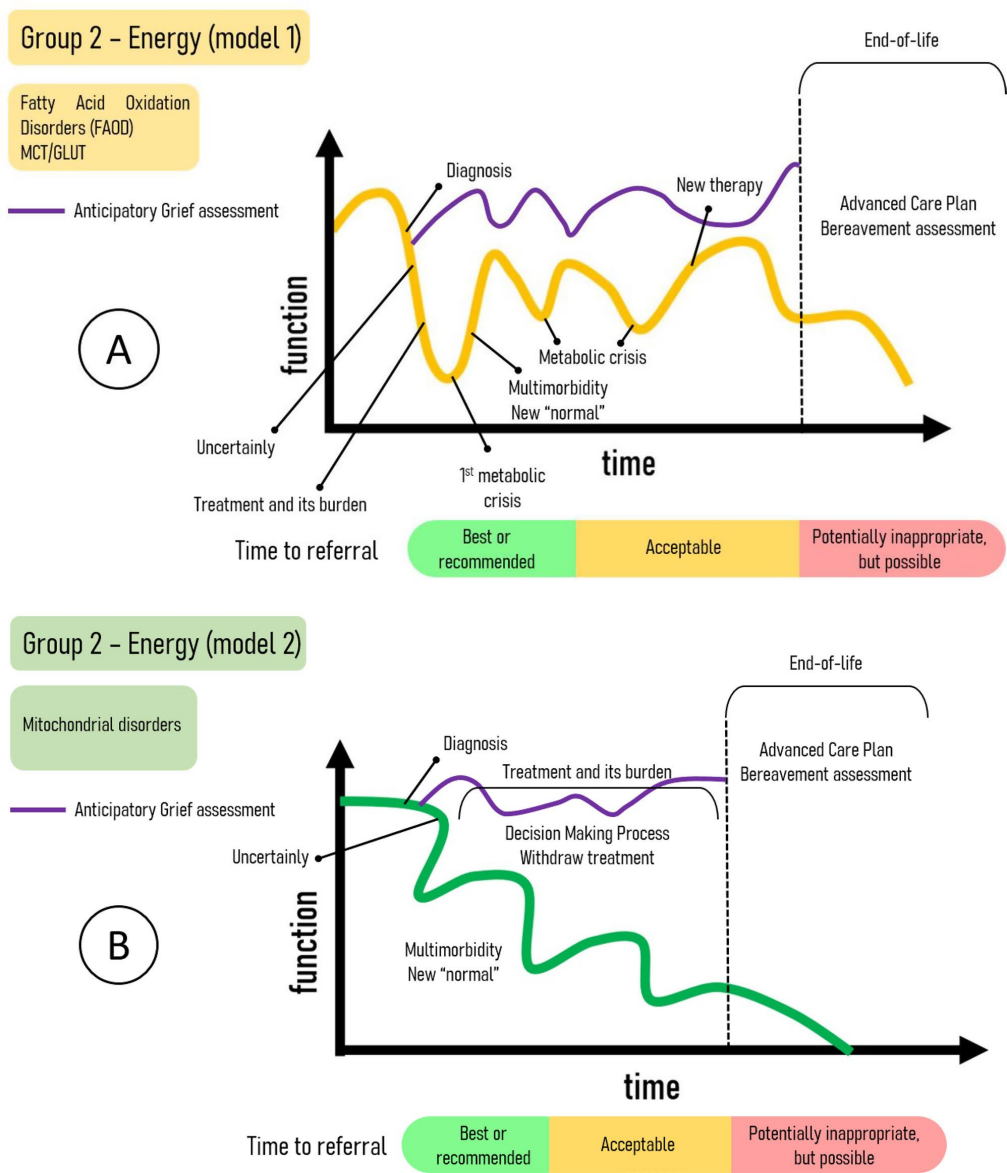
Trajectories of energy IMD (EIMD). **A** – fatty acid oxidation disorders and carriers defects can present stability moments with metabolic crises. **B** – Some presentations of mitochondrial disorders follow this trajectory

Group 2, comprising the energy disorders, also exhibits two distinct trajectory patterns. Category 1 includes fatty acid oxidation disorders (FAOD) and the disorders of energetic molecules carriers, such as monocarboxylate transporters (MCTs) and solute carriers (e.g. GLUT1). In contrast, Category 2 illustrates the most severe presentations of mitochondrial disorders. It is important to emphasize that not all mitochondrial disorders will follow the specific outcome depicted in Fig. 2-B.

**Fig. 3:**
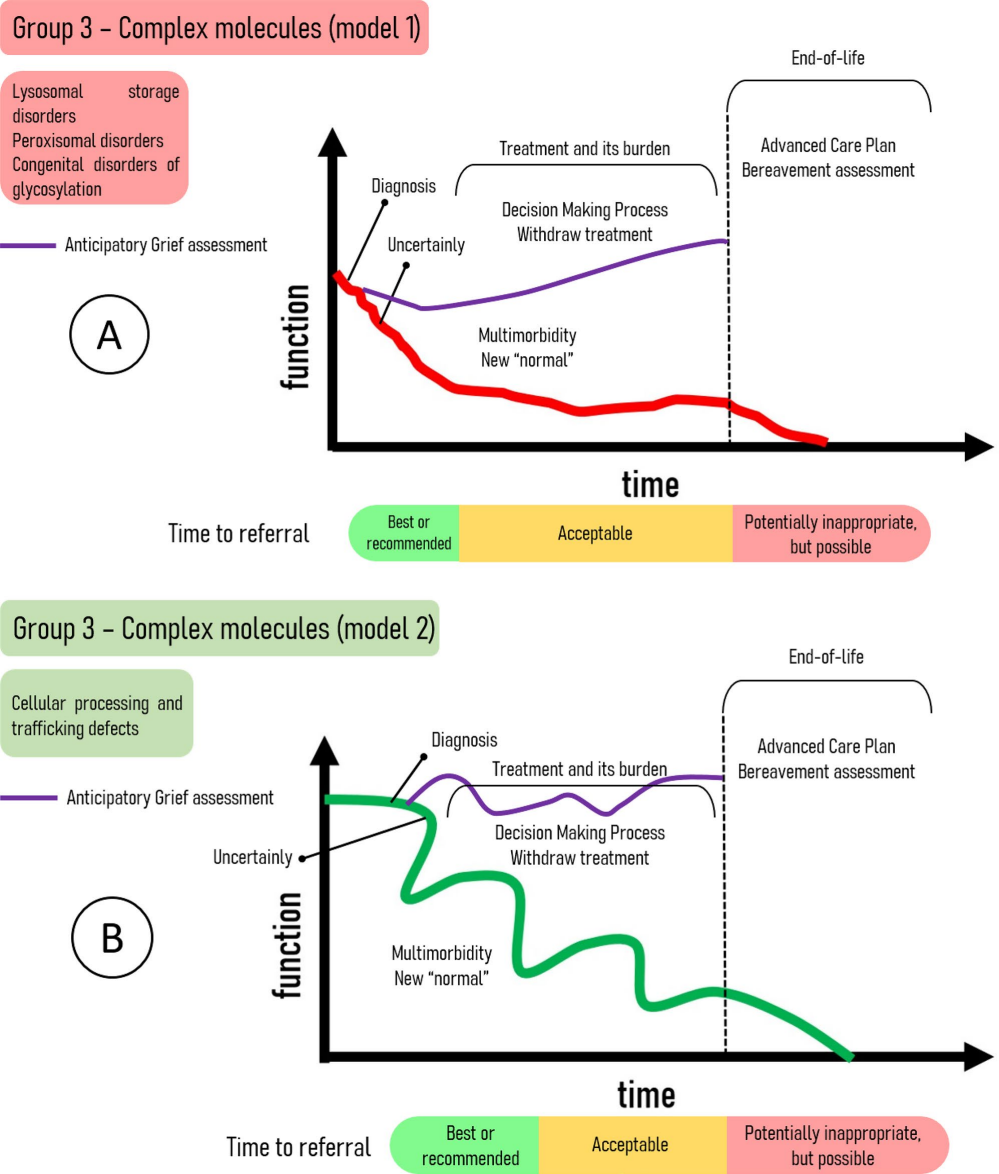
Trajectories of complex molecules disorders. **A** - Trajectories of storage disorders and congenital disorders of glycosylation, showing the earliest indication of PC referral. **B** – The outcomes of cellular processing and trafficking defects, the earliest group of IMD presenting unique diagnostic difficulties

Finally, the third pathophysiological group, characterized by the complex molecules disorders, presents two distinct trajectory categories. Category 1 primarily encompasses storage disorders and congenital disorders of glycosylation (CDGs). Category 2 comprises the more recently identified group of cellular processing and trafficking defects. Figure 3 shows these two trajectory categories, highlighting the proposed trigger points for initiating PC and a temporal scale indicating the optimal timing for referral to this specialty.

## Discussion

As previously stated, the integration of PC and IMD has encountered several barriers. However, a comprehensive perspective on adapting PC within the metabolic clinic can be derived from typical chronic illness trajectories. Key trigger points for initiating PC – such as the moment of diagnosis, uncertainty regarding disease progression, multimorbidity and end-of-life considerations – are common across all chronic complex diseases and will be discussed in detail below [8]. 

In the context of IMDs, additional factors are considered, including dietary management and its associated burden, metabolic crisis and the emergence of new therapies, particularly gene therapy. These specific considerations enhance the clarity of the referral timing and facilitate the personalization of PC for patients with IMD.

Finally, several gaps in the existing literature, even within the broader field of palliative care have been identified. These gaps include the decision-making process and the possibility of withdrawing treatments, which remain a nascent area involving complex bioethical issues. Additionally, the assessment of anticipatory grief is an underestimated topic in virtually all healthcare specialties.

### The diagnosis

The diagnosis of a serious illness is a pivotal event that profoundly impact a family’s life, marking the beginning of a new and often challenging trajectory [14]. For parents of children with intellectual disabilities, receiving a diagnosis is considered a highly significant event. It provides a framework for managing emotions, understanding available treatments, and making family planning decisions [15]. The emotional reaction of parents is deeply influenced by the diagnostic process itself, which can last anywhere from one to eight years, depending on the child’s clinical presentation, access to diagnostic tests, and the efficiency of the healthcare system – a journey often referred to as a “diagnostic odyssey” [16, 17].

Conversely, some families face the immense distress of an unknown diagnosis. This state of “not knowing” is a significant source of anxiety, fueled by future uncertainty, a sense of helplessness, and the inability to connect with families facing similar challenges. In this difficult context, effective communication is the most valuable tool for healthcare professionals to guide and support caregivers throughout the diagnosis process [17]. 

### The uncertainty

Following the diagnosis of an IMD, parents and caregivers are confronted with uncertainties throughout their lives. This uncertainty spans from the initial “diagnosis odyssey,” which can vary depending on a country’s diagnostics methods and access to technology, to the child’s long-term prognosis, including the risk of metabolic crises, the establishment of new clinical baselines, and responses to drug therapy.

An extensive body of literature documents the profound impact of an IMD diagnosis on parents. The most prevalent stressors identified include emotional, social, financial, and medical burdens, as well as a significant loss of personal freedom. Feelings of guilt and the unpredictable nature of symptoms and their triggers are also major sources of stress [4, 18, 19]. 

These caregivers must navigate a complex trajectory that involves coping with the “diagnostic odyssey”, understanding their child’s unique needs, adapting to changes in family dynamics, and overcoming challenges within the healthcare system [20]. 

### Dietary management and its burden

Throughout the diagnostic process and the subsequent uncertainty of the disease’s outcome, some inherited metabolic diseases (IMDs) require highly specific dietary management, which can be a significant source of stress for parents and caregivers. Recent studies have reinforced the substantial caregiver burden associated with strict dietary restrictions, particularly in organic acidurias (OA), urea cycle disorders (UCD), and maple syrup urine disease [21]. 

The impact of these dietary limitations on both patients and their caregivers has been a long- standing topic in the literature. These dietary requirements are often described as creating a significant social and emotional barriers, as they can be difficult to manage and communicate within a social environment [20]. 

### Metabolic crises

The fear of a metabolic crisis constitute a significant portion of the burden associated with dietary management. This constant threat directly impacts the health-related quality of life (HrQoL) for caregivers, who often live with a pervasive sense of strain, anxiety, and guilt related to a potential new episode [21]. 

For families of patients with intoxication-type inherited metabolic disease (IIMD) and fatty acid oxidation disorders (FAOD), the fear of a metabolic decompensation is a constant reality. This fear stems from the possibility of the loved one’s death or the onset of new symptoms and multimorbidity following an episode, which would lead to increased care requirements and greater dependence [19]. 

### Multimorbidity and the new normal

Navigating what can be described as“stranger tides” represents another significant challenge for these families. Following an acute decompensation episode, there is often the establishment of a new clinical *plateau*, or a “new normal”. This phenomenon became particularly evident during the COVID-19 pandemic.

Even in otherwise healthy children, the impact of an acute event on a caregiver’s functional status is substantial. This reflects how a child’s symptoms profoundly affect their daily life and family situation, leading to feeling of guilt, a sense of being lost, and the fear and hope associated with an uncertain future [22]. 

### Withdraw treatment

Despite limited discussion in the field of metabolic diseases, the withdrawal of treatments – particularly enzyme replacement therapies (ERT), is a critical issue that demands experience, technical knowledge, and a robust bioethical approach.

A parallel can be drawn with neuromuscular disorders, where the withdrawal of ventilatory support is discussed based on careful planning and communication, the promotion of patient and parental autonomy, and ongoing psychosocial and bereavement support for the family [23]. 

### Decision-making process

Defining the optimal treatment for a patient requires both a clinical and a bioethical perspective. This approach is facilitated through a decision-making process that involves open discussion of treatment options, living wills, personal values, and end-of-life (EOL) care preferences [24]. 

A four-step model has been proposed to guide the clinicians through the decision making process:

Step 1 (Ethics of Accuracy): Focuses on the disease, including its course, prognosis, and available treatments;

Step 2 (Ethics of Comprehension): Emphasizes patient biographies, values, perceptions of suffering, and treatment preferences;

Step 3 (Ethics of Situational Awareness): Involves clinical judgment and communication within the healthcare team, aiming to reconcile evidence-based treatments with the patient’s values and life history;

Step 4 (Ethics of Deliberation): Reinforces the patient-provider goals of care, ensuring the alignment between patient’s values and acceptable clinical practices [25]. 

A shared decision-making process is essential for optimizing patient care and promoting positive outcomes for both patients and their families.

### New therapies

Since the development of PKU formula in the mid-1950s, the treatment landscape for IMDs has expanded significantly. The last two decades, in particular, have seen notable advancements, including new drug therapies enabled by technological progress. Current therapeutic options for IMDs include dietary management, hematopoietic stem cell transplantation, enzyme replacement therapy (ERT), and solid organ transplantation [26]. 

Gene therapy has also emerged as a promising avenue within the metabolic field, offering hope for improved patient outcomes and enhanced quality of life [27]. 

Despite these advancements, a common misconception exists among some healthcare professionals that palliative care (PC) is antithetical to disease-targeted treatment. On the contrary, palliative care works in conjunction with curative or life-prolonging therapies. It operates on the understanding that the burden of illness stems not only from the disease itself but also from its demanding treatment protocols. Therefore, the integration of PC is justified and beneficial regardless of the treatment outcome [28]. 

### End-of-life

The existing literature lacks comprehensive articles on the end-of-life process for patients with IMD, particularly concerning advanced care planning, the most prevalent symptoms, and bereavement support.

Despite this gap, it is well-established that engaging patients and their families in advance care planning through a shared decision-making process yields significant benefits. These include increased death preparedness, less aggressive medical interventions, and an improved quality of life for patients. For families, this approach leads to better bereavement outcomes post-loss, reduced decision burden and lower psychological distress [29]. 

Furthermore, these benefits underscore the importance of conceptualizing palliative care as a *continuum*. PC extends beyond the patient’s death to provide ongoing support, including structured bereavement follow-up for the family [1]. 

### Anticipatory grief assessment

A concept that is rarely discussed in the context of bereavement is anticipatory grief (AG). It is defined as the emotional and psychological reaction of caregivers to the perceived multiple losses experienced by their loved ones with a life-threatening illness, particularly as they approach death [30]. 

First proposed in 1955 based on observations of mothers of children with leukemia, the concept originally linked parental and child anxieties to the fluctuating course of the illness [31]. In parents of fatally ill child, grief may begin immediately upon diagnosis, continue throughout the child’s disease trajectory, and persist after the child’s death [32]. 

Over seventy years after its initial description, AG has been quantified, with its presence noted in approximately 25% of caregivers of individuals with a life-threatening illness. The cumulative burden of care and the personal losses contributes to a range of mental, physical and social challenges, such as anxiety, guilt, shame and fatigue [30]. 

AG is also a source of distress and an ambivalent experience, often stemming from the anticipation of death and the secondary losses associated with the patient’s physical decline and increasing care needs [33]. 

## Conclusion

Establishing a visual framework of trigger points for PC assessment provides metabolic clinicians with a structured method to identify opportunities for partnership. This collaborative approach enhances patient care by addressing the multifaceted burdens of complex chronic diseases on both patients and their caregivers. By demystifying and facilitating early referral to PC, clinicians can ensure that the patient remains the central focus throughout their illness trajectory.

## Data Availability

No clinical data were used in this article.
